# Innovations in Lung Transplant Research and Practice: The Future is Now

**DOI:** 10.3389/ti.2025.14336

**Published:** 2025-02-04

**Authors:** Robin Vos, Sandra Lindstedt, Deborah J. Levine, Norihisa Shigemura

**Affiliations:** ^1^ Department of Respiratory Diseases, University Hospitals Leuven, Leuven, Belgium; ^2^ Department Chronic Diseases, Metabolism and Ageing (CHROMETA), Laboratory of Respiratory Diseases and Thoracic Surgery (BREATHE), KU Leuven, Leuven, Belgium; ^3^ Wallenberg Centre for Molecular Medicine, Lund University, Lund, Sweden; ^4^ Department of Clinical Sciences, Lund University, Lund, Sweden; ^5^ Lund Stem Cell Centre, Lund University, Lund, Sweden; ^6^ Department of Cardiothoracic Surgery and Transplantation, Skåne University Hospital, Lund, Sweden; ^7^ Division of Pulmonary, Critical Care and Allergy, Stanford University, Palo Alto, CA, United States; ^8^ Cardiothoracic Surgery, University of Pittsburgh School of Medicine, Pittsburgh, PA, United States

## Introduction

Recent advancements in transplantation research and practice have focused on expanding the donor pool, developing novel biomarkers and diagnostic tools, and exploring innovative treatment opportunities. These efforts aim to address the persistent shortage of donor organs and improve long-term outcomes for transplant recipients.

With the coming of age of artificial intelligence (AI) in the 21st Century, it is expected that in the next decade medical transplant research - and its clinical implications - will exponentially lead to new discoveries, deepened insights, and better management of the logistical processes and disease mechanisms involved in transplantation. However, surprisingly none of the research papers included in our special issue of Transplant International on “*Lung transplantation in the 21st Century: innovative care for improved outcomes*” implemented the use of AI methodologies in their research. It is nevertheless expected that the “old-school” approach of performing scientific research is to be replaced by research practices incorporating AI tools to analyze, visualize, summarize, and present research findings. Is this a bad evolution? Probably - and hopefully - not, as proper use of AI may help to galvanize big data and complex research findings into more efficient logistical flows, better diagnostics, novel therapies, and more personalized treatment options in the field of transplantation.

The authors of the current editorial therefore opted to use a freely available AI tool (Perplexity AI, Inc., San Francisco, United States) to synthesize information regarding the sixteen scientific papers in the field of transplantation which were included in this special issue of Transplant International. The use of AI in medical reporting demonstrates its potential to efficiently aggregate and distill complex scientific information, potentially accelerating the dissemination of knowledge in rapidly evolving fields like transplantation medicine. However, it is crucial to note that AI-generated summaries should be reviewed and validated by subject matter experts to ensure scientific accuracy and contextual relevance. Post-generation, this summary was therefore manually checked and edited by the authors.

### Expanding the Donor Pool

#### Organ Preservation and Reconditioning

Considerable progress has been made in organ preservation techniques, moving beyond traditional static cold storage. Hoetzenecker et al. introduced the concept of semi-elective lung transplantation using prolonged static storage at 10°C, potentially expanding the geographical range for organ allocation [1]. The authors highlight that this method allows for prolonged cold ischemic times, up to 24 h, without compromising organ function or short-term outcomes. This technique has been validated through large animal experiments and a multi-center observational study. The 10°C preservation temperature has shown to cause less mitochondrial damage compared to traditional ice storage methods. This development could potentially transform lung transplantation logistics, enabling semi-elective procedures and improving organ sharing between distant regions.

Cenik et al. reviewed the principles of controlled hypothermic storage (CHS) for lung preservation in transplantation [2]. CHS allows preservation at temperatures higher than traditional ice storage, typically around 10°C. Animal experiments showed superior lung physiology after prolonged storage at 10°C compared to ≤4°C. Molecular analyses revealed better protection of mitochondrial health and higher levels of antioxidative metabolites with CHS. While initial clinical findings are promising and suggest a need for withdrawal from conventional ice-based method, further research is needed to draw more robust conclusions about the safety and efficacy of CHS in lung transplantation.

These studies highlight that novel organ preservation techniques may nevertheless help increase the number of viable donor organs and improve transplant outcomes soon.

*Ex vivo* lung perfusion (EVLP) has emerged as a promising technique for organ reconditioning. Chilvers et al. developed a split-lung *ex vivo* perfusion model, allowing for time- and cost-effective evaluation of therapeutic interventions in human donor lungs [3]. Their split-lung EVLP model that allows for the simultaneous perfusion and ventilation of two single lungs from the same donor. This offers several advantages: i) it provides a cost-effective and reliable platform for testing therapeutic interventions on human donor lungs, ii) the split-lung approach allows one lung to receive an intervention while the other serves as a control, eliminating inter-donor variation, iii) the model facilitates continuous monitoring of hemodynamic and airway parameters, as well as blood gas, perfusate, and tissue sampling, iv) pulmonary edema can be assessed directly using ultrasound and indirectly through lung tissue wet:dry ratio measurements, and v) this approach enables researchers to evaluate promising interventions more efficiently, potentially increasing the number of transplantable organs.

This new EVLP model could therefore accelerate research into organ preservation and reconditioning strategies.

#### Expanding Controlled Donation After Circulatory Determination of Death

Moreno et al. reported on lung transplantation in controlled donation after circulatory determination of death (cDCD) using abdominal normothermic regional perfusion (A-NRP) [4]. The authors present an update on cDCD lung donation, highlighting its potential to alleviate the shortage of transplantable lungs. They describe the Maastricht classification of DCD donors and emphasize that cDCD is the most accepted type for lung donation. The paper includes a step-by-step protocol for lung procurement using A-NRP, which is critical for achieving high retrieval rates. The authors also discuss donor selection criteria and the importance of adequate management in the intensive care unit.

This study highlights that increased use of cDCD with A-NRP may expand the donor pool.

Sandiumenge et al. compared systemic inflammation in brain dead (DBD) and DCD lung donors and its impact on lung transplant recipients [5]. The researchers measured plasma levels of cytokines IL-6, IL-8, IL-10, and TNF-α in 40 DBD and 40 DCD donors and their recipients. Results showed significantly higher levels of IL-6, IL-10, and IL-8 in DBD donors compared to DCD donors. Higher TNF-α levels in donors were associated with a higher incidence of primary graft dysfunction (PGD) in recipients.

The study highlights that DBD is associated with higher systemic inflammation than DCD, and higher donor TNF-α levels correlate with increased PGD incidence, which results may allow for tailored anti-inflammatory treatments to attenuate PGD, potentially improving patient outcomes.

#### Organ Allocation Practices

Shudo et al. evaluated the impact of the revised United Network for Organ Sharing heart allocation policy implemented in October 2018 on en-bloc heart-lung transplantation outcomes [6]. The researchers analyzed data from adult patients registered for heart-lung transplants before and after the policy change. Results showed significantly higher transplant rates, shorter waitlist times, and reduced waitlist mortality in the post-policy period. Despite higher-risk recipients in the post-period, short-term survival rates remained similar before and after the policy change.

The study highlights that the revised policy significantly improved access to en-bloc heart-lung allografts with better waitlist outcomes and similar post-transplant outcome.

### Biomarkers and Diagnostic Tools

#### Donor-Derived Cell-Free DNA as Biomarker for Rejection

Novo et al. investigated the potential of cell-free DNA (cfDNA) as a non-invasive biomarker for predicting complications after lung transplantation [7]. The researchers analyzed 246 serum samples from 26 lung transplant recipients, focusing on chronic lung allograft dysfunction (CLAD). They used four different methods to measure donor fractions of cfDNA, including three digital droplet PCR applications and one method measuring absolute amounts of donor-derived cfDNA. The results showed statistically significant elevations of cfDNA in CLAD samples compared to non-CLAD samples across all four methods.

This study highlights the use of digital droplet PCR-detected cfDNA as a potential biomarker for predicting CLAD and differentiating rejection from infection in lung transplant recipients.

#### Immunologic Biomarkers of Rejection

Auner et al. studied the clinical significance of transient and persistent *de novo* donor-specific antibodies (dnDSAs) in lung transplantation [8]. The researchers analyzed 405 lung transplant recipients, of whom 205 developed dnDSAs. They found that persistent, but not transient, dnDSAs were associated with CLAD and antibody-mediated rejection (AMR). Patients with persistent dnDSAs had significantly lower CLAD-free survival rates at 1-, 3-, and 5-year post-transplantation compared to those with transient dnDSAs.

The study highlights the importance of distinguishing between transient and persistent dnDSAs in predicting outcomes after lung transplantation and may guide future management, suggesting the need for prompt treatment of persistent dnDSAs.

Zajacova et al. compared histological and molecular diagnoses of lung transplant rejection, focusing on treatment responses [9]. The researchers analyzed 54 transbronchial biopsies from lung transplant recipients between 2015 and 2020. They found discrepancies between histological and molecular diagnoses in 54% of cases. Patients with molecular T-cell mediated rejection (TCMR) showed a significantly higher treatment response rate (50%) compared to those with no rejection (14%).

The study findings suggest that low-grade acute cellular rejection (ACR) may not always correspond with molecular TCMR, indicating that molecular diagnosis could better identify patients who would benefit from anti-rejection therapy.

Novysedlak et al. identified elevated PD-L1 and PECAM-1 as potential diagnostic biomarkers of ACR in lung transplantation [10]. The researchers observed a significant increase in PD-L1 tissue expression within the ACR group, suggesting an attempt to suppress immune responses. PECAM-1 levels were also elevated in cases of ACR. The findings indicate that both PD-L1 and PECAM-1 could serve as valuable markers for diagnosing ACR in lung transplant recipients.

This research may contribute to the development of more accurate diagnostic tools for identifying rejection in lung transplantation, potentially improving patient outcomes.

### Treatment Opportunities

#### Peri-Operative Considerations

Vaiter et al. investigated the effects of lower doses of unfractionated heparin (UFH) for intraoperative extracorporeal membrane oxygenation (ECMO) anticoagulation [11]. The researchers analyzed 109 lung transplant patients who underwent central VA ECMO support between 2020 and 20223. They found that lower UFH doses led to reduced intraoperative blood derivative consumption and blood loss without increasing thrombotic complications. The study also suggests that lower UFH doses may decrease the incidence of surgical revision for hemothorax.

The study highlights that using lower doses of UFH for intraoperative ECMO anticoagulation during lung transplantation might reduce complications and lead to better outcomes.

Li et al. investigated risk factors, incidence, and outcomes associated with clinically significant airway ischemia (CSAI) in lung transplant recipients [12]. The researchers reviewed 217 lung transplants performed between 2016 and 2020, finding that 37.8% of patients developed CSAI. Risk factors for CSAI included recipient diabetes, intraoperative ECMO use, and single running suture technique. Patients with CSAI, particularly those who developed dehiscence or stenosis, had lower survival rates compared to those without CSAI.

The study highlights the importance of mitigating risk factors, identifying and managing CSAI to improve outcomes in lung transplant recipients.

Palleschi et al. reviewed the complex relationship between the diaphragm and lung transplantation [13]. The authors discuss how several factors before transplantation, including underlying respiratory diseases and comorbidities, can impact diaphragmatic function. They highlight that the surgical procedure itself can cause trauma to the diaphragm, potentially leading to morphological and functional alterations. Conversely, the diaphragm influences aspects of lung transplantation, from graft-to-chest cavity size matching to long-term postoperative respiratory performance.

The review emphasizes the need for careful dissection during the lung transplant procedure to avoid trauma to the phrenic nerve and diaphragm, but also the lack of standard criteria for evaluating and managing diaphragmatic dysfunction in lung transplantation, which hinders accurate assessment of outcomes.

Sempere et al. investigated systemic absorption of inhaled tobramycin in lung transplant recipients [14]. The researchers conducted a retrospective analysis of adult patients treated with inhaled tobramycin for at least 3 days. The primary indications for treatment were donor bronchial aspirate bacterial isolation (18 patients) and tracheobronchitis (15 patients). Key findings include: i) 82% of patients had detectable serum tobramycin levels, with 26% showing elevated levels (>2 μg/mL), ii) 26% of patients developed acute kidney injury during treatment, and iii) invasively mechanically ventilated patients had significantly higher median trough tobramycin levels compared to non-ventilated individuals.

The study concludes that inhaled tobramycin administration in lung transplant recipients, especially in those on invasive mechanical ventilation, may result in substantial systemic absorption, which is important to consider in the early post-transplant phase.

#### Immunomodulation and Tolerance Induction

Jin et al. reviewed the use of donor-specific blood transfusion (DSBT) in lung transplantation as a potential strategy for inducing immunological tolerance [15].

DSBT involves infusing fresh whole blood from the donor to the recipient before transplantation, aiming to improve graft acceptance and potentially induce donor-specific tolerance. The review summarizes existing knowledge on DSBT mechanisms and outcomes in solid organ transplants, including preclinical and clinical settings. It explores associations with regulatory T cells, mononuclear phagocytic cell modulation, and microchimerism. The authors also discuss potential benefits and risks of DSBT in lung transplantation, offering insights for future research directions.

The review highlights that this approach, if successful, could help reduce the need for long-term immunosuppression and its associated complications.

Messika et al. reviewed the diagnosis and therapeutic armamentarium for AMR in lung transplantation [16]. The authors highlight the importance of identifying DSA and their association with various forms of rejection. The review explores current diagnostic methods and therapeutic approaches for AMR, including desensitization techniques and targeting the complement cascade. It also emphasizes the use of combined strategies such as immune cell depletion, immune pathway inhibition, and inflammatory cascade modulation.

The review highlights that these innovative techniques offer promising perspectives for lung transplant recipients facing this challenging complication.

#### Stem Cell Therapies, Regenerative Medicine, and Xenotransplantation

While not explicitly covered in the provided papers, stem cell therapies, regenerative medicine and xenotransplant approaches represent promising avenues for future research and treatment practices in transplantation, potentially offering new ways to repair or replace damaged organs.

## Conclusion

In conclusion, recent advances in transplantation research and practice demonstrate a multifaceted approach to addressing the challenges of organ shortage and improving long-term outcomes, with promising developments in organ preservation, biomarker discovery, and immunomodulation strategies. Also, the use of AI in medical reporting has an immense potential to efficiently aggregate and distill complex scientific information, as demonstrated herein. Moreover, incorporation of AI tools in research methods may radically shape the 21st Century of (lung) transplant medicine, as is already evidenced by the steep increase in the number of scientific publications reporting on AI in transplant research since 2020 [17]. Hence, in the coming years AI is expected to truly transform our field by turning (pre-)clinical data into innovative care that results in improved outcomes of our transplant patients. So, let the future begin!

## Abbreviations

A-NRP, abdominal normothermic regional perfusion; ACR, acute cellular rejection; AI, artificial intelligence; AMR, antibody-mediated rejection; cfDNA, cell-free DNA; CHS, controlled hypothermic storage; CSAI, clinically significant airway ischemia; CLAD, chronic lung allograft dysfunction; DBD, donation after brain death; DCD, donation after circulatory determination of death; dnDSAs, *de novo* donor-specific antibodies; DSBT, donor-specific blood transfusion; ECMO, extracorporeal membrane oxygenation; EVLP, *ex vivo* lung perfusion; PGD, primary graft dysfunction; TCMR, T-cell mediated rejection; UFH, unfractionated heparin.
